# CYP709B3, a cytochrome P450 monooxygenase gene involved in salt tolerance in *Arabidopsis thaliana*

**DOI:** 10.1186/1471-2229-13-169

**Published:** 2013-10-28

**Authors:** Guohong Mao, Timothy Seebeck, Denyse Schrenker, Oliver Yu

**Affiliations:** 1 Donald Danforth Plant Science Center, 975 North Warson Road, St. Louis, MO 63132, USA; 2 Present address: Conagen Inc., 1005 North Warson Road, St., Louis, MO 63132, USA; 3 Present address: The Pennsylvania State University, 115 Agricultural Sciences and Industries Building, University Park, PA 16802, USA

**Keywords:** CYP709B, Salt tolerance, Germination, Expression pattern, Metabolic profile

## Abstract

**Background:**

Within the *Arabidopsis* genome, there are 272 cytochrome P450 monooxygenase (P450) genes. However, the biological functions of the majority of these P450s remain unknown. The CYP709B family of P450s includes three gene members, *CYP709B1*, *CYP709B2* and *CYP709B3*, which have high amino acid sequence similarity and lack reports elucidating biological functions.

**Results:**

We identified T-DNA insertion-based null mutants of the CYP709B subfamily of genes. No obvious morphological phenotypes were exhibited under normal growth conditions. When the responses to ABA and salt stress were studied in these mutants, only the *cyp709b3* mutant showed sensitivity to ABA and salt during germination. Under moderate salt treatment (150 mM NaCl), *cyp709b3* showed a higher percentage of damaged seedlings, indicating a lower tolerance to salt stress. *CYP709B3* was highly expressed in all analyzed tissues and especially high in seedlings and leaves. In contrast, *CYP709B1* and *CYP709B2* were highly expressed in siliques, but were at very low levels in other tissues. Under salt stress condition, *CYP709B3* gene expression was induced after 24 hr and remained at high expression level. Expression of the wild type CYP709B3 gene in the *cyp709b3* mutant fully complemented the salt intolerant phenotype. Furthermore, metabolite profiling analysis revealed some differences between wild type and *cyp709b3* mutant plants, supporting the salt intolerance phenotype of the *cyp709b3* mutant.

**Conclusions:**

These results suggest that *CYP709B3* plays a role in ABA and salt stress response and provides evidence to support the functions of cytochrome P450 enzymes in plant stress response.

## Background

Cytochrome P450 monooxygenases (P450s) are universal enzymes present in most organisms from bacteria to plants and humans. They catalyze the oxidation of various substrates through activation of molecular oxygen. All P450s share a common catalytic center; a heme with an iron coordinated to the thiolate of a conserved cysteine. In plants, a large number of P450 genes form a superfamily and play important roles in plant metabolic processes [1-3].

Plant P450s are generally classified into two main clades: the A-type and the non-A-type. The A-type clade is specific to plants while the non-A-type clade is a divergent group with members showing more similarity to non-plant P450s [4,5]. Plant P450s catalyze biosynthetic steps for a wide range of plant metabolites, including pigments, defense-related compounds, UV protectants, lignin, fatty acids and phytohormones [2,3,6-8].

Plant genomes encode much higher numbers of P450 genes than those of other organisms, reflecting the broad biological functions of P450s in primary and secondary metabolism. In the *Arabidopsis thaliana* genome, the P450 superfamily contains 272 genes (including 26 pseudogenes), of which 153 are A-type P450s and 93 are non-A-type P450s [3]. Thus far a small number of the 246 putative coding sequences have been associated with a specific biochemical function [3,7], meaning that the biological functions of the majority of the P450 genes in *Arabidopsis* remain unknown.

As new technical approaches are developed, the biological functions of more plant P450s have been identified. Expression and co-expression analysis provides clues for functional annotation of P450s in *Arabidopsis*. For example, Narusaka et al. studied the crosstalk between abiotic and biotic stress responses using a cDNA microarray containing the genes in the cytochrome P450 superfamily [9]. The Werck-Reichhart group generated an extensive co-expression analysis tool for the cytochrome P450 superfamily [10,11]. These expression analyses provided novel clues to the functions, metabolic pathways and regulatory networks of individual P450s. Also, based on co-expression analysis, a novel phenolic pathway in pollen development was identified [12]. An abundance of P450 expression data was collected that provides information into the biological functions of P450s in plant development and the responses to chemical and environmental stresses. For example, the P450s CYP71A19, CYP71B19, CYP71B20, CYP71B26, CYP71B28, CYP76C2, CYP86B1, CYP89A9 and CYP94B3 are induced in response to ABA treatments (3 to 24 h), IAA treatment (3 h) and osmotic stress (3 h); patterns similar to those of CYP707A1, which is known to mediate ABA catabolism [7,13]. These data are starting points to identify the functions of these P450s in stress response.

Heterologous or *in vitro* expression has proven a useful tool in unveiling the biochemical functions of P450s. For instance, CYP735A1 and CYP735A2 were identified as cytokinin hydroxylases that catalyze the biosynthesis of *trans*-Zeatin by using an *adenosine phosphate-isopentenyltransferase* (*AtIPT4*)*/P450* co-expression system in yeast [14]. In addition, the CYP707A1-CYP707A4 genes were functionally expressed in yeast and found to have an ABA 8′-hydroxylase activity [15]. Despite these successes, the membrane-bound nature of P450 proteins and the presence of P450 reductases create special challenges for using heterologous systems, and thus have limited the number of P450s with defined enzymatic activity.

Some stress related plant P450 genes were identified by genetic screening [7,8]. For example, *cyp707a* mutants exhibited hyperdormancy in seeds and accumulated greater ABA content than wild type, indicating that CYP707 genes regulate ABA catabolism [15-18].

At present, there is limited information about the CYP709B subfamily. Expression data showed that some of the *CYP709B* genes were regulated by phytohormones [19] and circadian rhythm [20]. No enzymatic activity was identified by using the yeast expression system [14,21,22]. In this report, using genetic screening, we identified the null mutants of the CYP709B genes and compared the phenotypes in germination and salt tolerance. Only the *cyp709b3* mutant exhibited the ABA and salt sensitive phenotypes. Expression of the wild type CYP709B3 gene in the *cyp709b3* mutant fully complemented the salt intolerance phenotype. The possible function of CYP709B3 in salt tolerance is also discussed.

## Results

### Identification of T-DNA insertion mutants of CYP709B family genes

The CYP709B subfamily belongs to non-A-type cytochrome P450s and includes three gene members: *CYP709B1*, *CYP709B2* and *CYP709B3*. The putative proteins share high identity at the amino acid level (Additional file 1). The *CYP709B1* (At2g46960) and *CYP709B2* (At2g46950) genes are located on chromosome 2 and both have 5 exons and 4 introns. Physically, they are 759 bp apart in genomic sequence. *CYP709B3* (At4g27710) is located on chromosome 4 and also has 5 exons and 4 introns.

**Figure 1:**
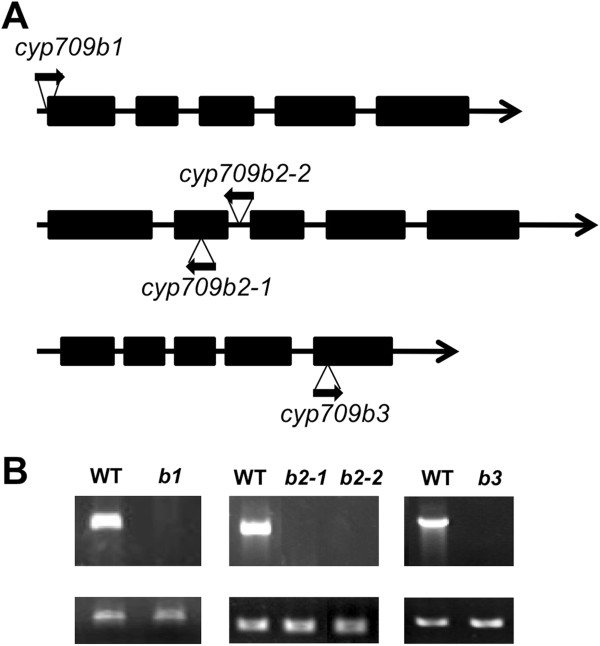
**Identification of CYP709B subfamily mutants from T-DNA mutant collections. A.** Structure of the CYP709B family genes showing the positions of the T-DNA insertions in mutants. **B.** Levels of transcripts in wild-type (WT) and mutants were determined by RT-PCR. WT: wild type; *b1*: *cyp709b1*; *b2-1*: *cyp709b2-1*; *b2-2*: *cyp709b2-2*; *b3*: *cyp709b3*. The *ACTIN2* transcript level was used as a control (bottom panel).

We identified T-DNA insertion mutants in each of the CYP709B subfamily members, all in Columbia-0 (Col-0) background. SALK_021290C (*cyp709b1*) has an insertion in the promoter of the *CYP709B1* gene, and SALK_011121 (*cyp709b3*) has an insertion in the fifth exon of the *CYP709B3* gene. We identified two mutant alleles of the *CYP709B2* gene, one of which has an insertion in the second exon (SALK_020401, *cyp709b2-1*) and the other in the third intron (SALK_087806, *cyp709b2-2*) (Figure 1A). Reverse transcription RT-PCR analysis of the total RNA from mutant leaves or flowers was performed using gene-specific primers. As shown in Figure 1B, the gene transcripts were not detected in the mutants, demonstrating that all mutants were transcript null.

### Tissue-specific expression pattern of CYP709B subfamily genes

**Figure 2:**
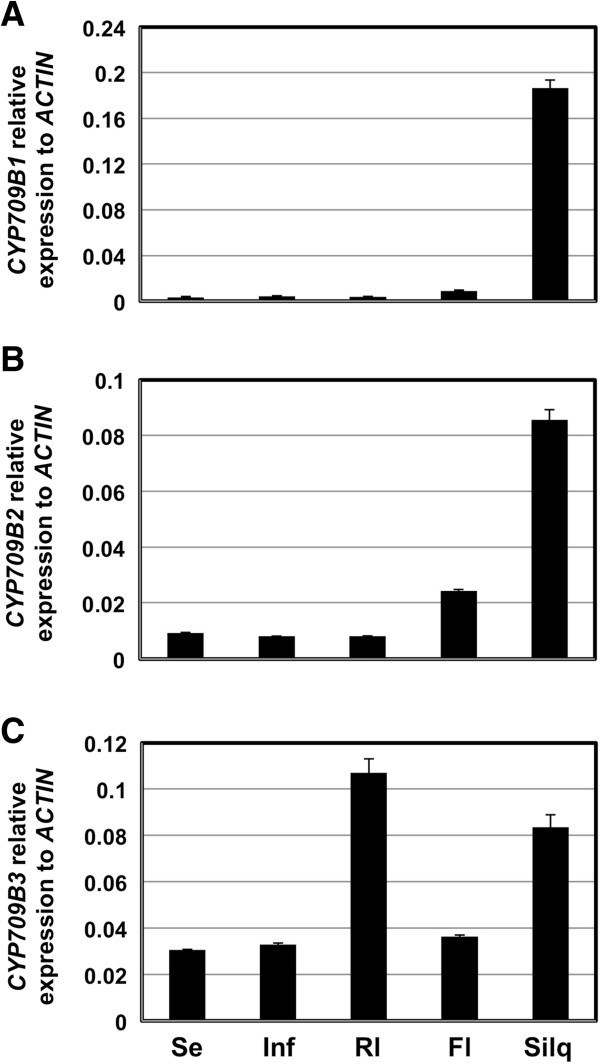
**Tissue-specific expression in wild type*Arabidopsis*. A:***CYP709B1*. **B:***CYP709B2.***(C)***CYP709B3*. Se: 12-day-old seedlings; Inf: inflorescences; Rl: rosette leaves; FL: opening flowers; Silq: siliques (4–10 DAP). Real time PCR was performed to detect the level of transcripts in various organs of wild type. *ACTIN2* was used as internal control. Values are the means ± SE of three replicates.

The transcript levels of the *CYP709B* genes from seedlings, inflorescences, rosette leaves, flowers and siliques were analyzed by a quantitative real time PCR method. *CYP709B1* and *CYP709B2* showed very low expression levels in seedlings, inflorescences, and rosettes, but had high expression levels in siliques (Figure 2A and B). Transcripts of *CYP709B3* were detected in all tested organs and were more abundant in rosette leaves and siliques (Figure 2C). Recently, a batch of microarray data also showed similar expression patterns (http://bbc.botany.utoronto.ca)[23]. As confirmed by our data and by available database information, the *CYP709B3* gene is universally expressed while the *CYP709B1* and *CYP709B2* are highly expressed in mature siliques, indicating that these closely related genes may have different biological functions.

### *cyp709b3* mutant is sensitive to ABA and salt stress in germination

**Figure 3:**
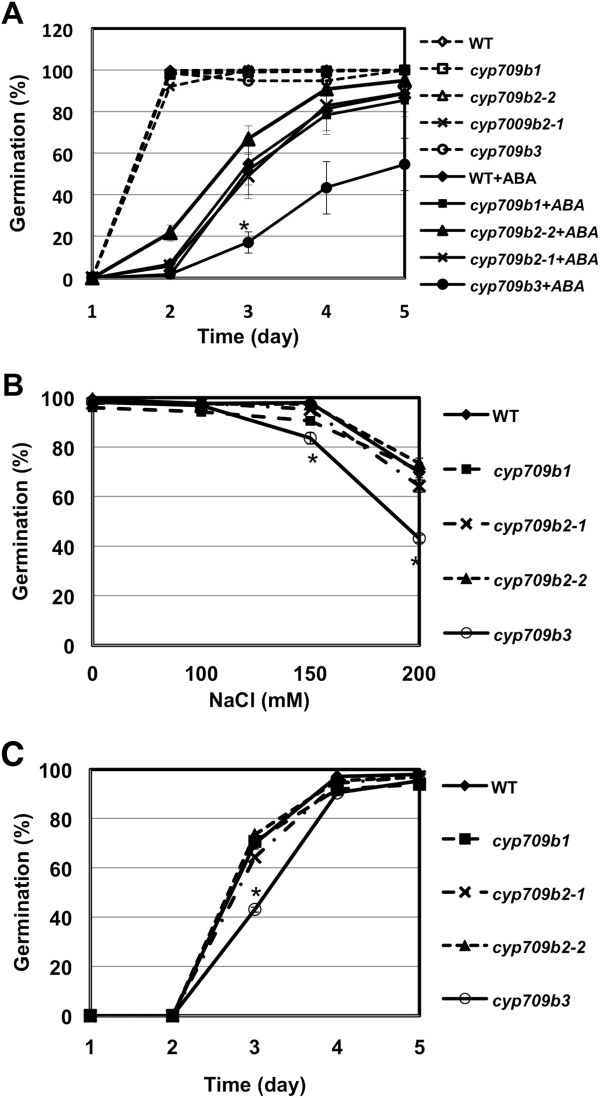
**Phenotypic analysis of*cyp709b1*,*cyp709b2*and*cyp709b3*germination. A.** Germination of *cyp709b3* is hypersensitive to ABA. Seeds were sown on wetted filter paper containing 0 and 1.5 μM ABA. After 2 days at 4°C, the plates were placed under continuous light. Germination (emergence of radicals) was scored at indicated times. **B-C.** Germination of *cyp709b3* is sensitive to salt treatment. Seeds were sown on MS plates with 0, 100, 150 or 200 mM NaCl. Germination was scored at the indicated times after 2 days cold (4°C) treatment. **B:** Germination at day 3 under different NaCl conditions; **C:** Germination under 200 mM NaCl at indicated time. Error bars indicate SE (n = 3). Statistically different to wild type (p value < 0.05) is indicated using asterisks.

The *CYP709B* T-DNA insertion mutants displayed no visible phenotypic alterations under normal growth conditions. The mature plant height, rosette size, flower, silique and seed were all similar to wild type, suggesting that the *CYP709B* genes are not essential to vegetative or reproductive growth and development. Since all three genes were highly expressed in siliques and seeds, we speculated that loss-of-function of the *CYP709B* genes would lead to seed-related phenotypes, such as in seed dormancy and/or germination. To assess whether any of the CYP709B genes play roles in controlling seed germination, we performed germination assays using wild type and *cyp709b* mutants. To determine whether the mutation affects seed germination in response to ABA, wild type and mutant seed was sown on filter paper saturated with water and different concentrations of ABA. After stratification at 4°C for 2 days, germination was scored daily. Without ABA treatment, all seeds germinated; reaching around 100% at day 2. Germination of the *cyp709b3* mutant was inhibited by application of ABA (Figure 3A). In contrast, seed of the other two mutants germinated similarly to wild type under ABA treatment. In the presence of 1.5 μM ABA, the germination of *cyp709b3* was dramatically delayed. At day 5, around 90% of the wild type seeds had germinated, while only 55% of the *cyp709b3* seeds had. The delayed germination in *cyp709b3* seeds was presumably due to increased sensitivity to exogenous ABA.

*cyp709b3* seed germination was also more sensitive to salt stress than seeds of wild type and the other mutants. In the presence of 150 or 200 mM NaCl, the germination of *cyp709b3* seeds was delayed. At day 3, only 83% and 43% of the *cyp709b3* seeds germinated in the presence of 150 and 200 mM NaCl, respectively. In contrast, 98% and 70% of the wild type seeds had germinated. The *cyp709b2-1*, *cyp709b2-2* and *cyp709b1* mutant seeds exhibited similar germination rates as wild type (Figure 3 B and C). Although the germination of *cyp709b3* seeds was inhibited at day 3 under 200 mM NaCl treatment, it was similar to the wild type germination rate at day 4. These results indicate that only the *cyp709b3* mutant is sensitive to ABA and salt during germination.

### *cyp709b3* mutant shows a salt intolerance phenotype

**Figure 4:**
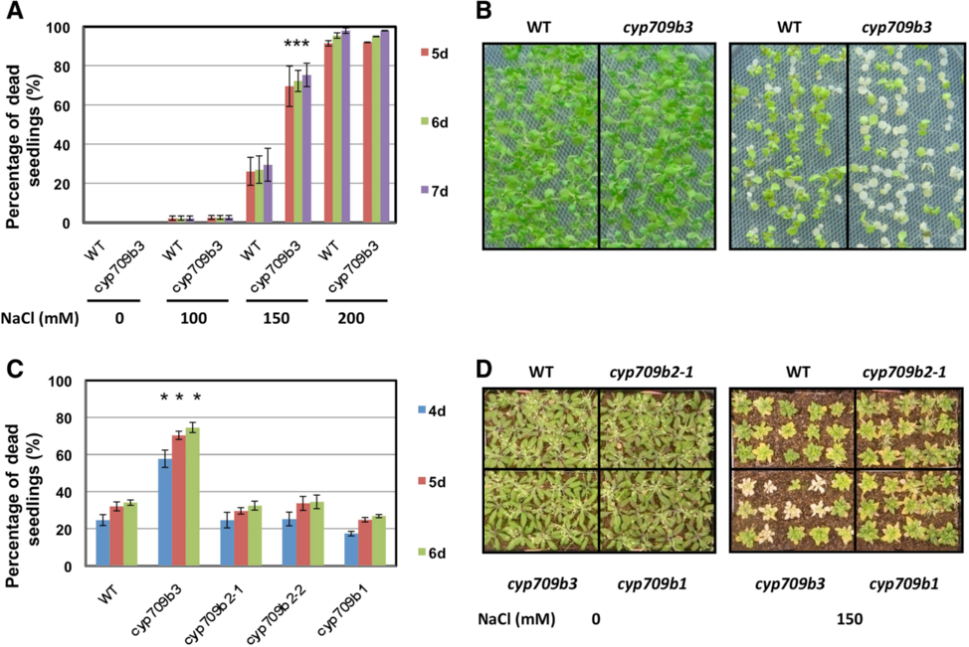
**The*cyp709b3*mutant plant is sensitive to salt stress. A.** Rate of dead seedlings at day 5, 6 and 7 after transfer onto NaCl agar plates. Seeds were germinated on MS medium for 4 days and transferred onto MS agar plates supplemented with 0, 100, 150 or 200 mM NaCl. **B.** Growth of WT and *cyp709b3* seedlings on MS medium (left) and medium supplemented with 150 mM NaCl (right) at day 5 after transfer. **C.***cyp709b1* and *cyp709b2* mutant seedlings are not sensitive to salt stress. **D.** Growth of WT and *cyp709b1*, *cyp709b2-1* and *cyp709b3* mutant plants in soil. Seedlings were irrigated with water (left) or 150 mM NaCl (right) after 21 days. These pictures were taken at day 21 after treatment initiation. Error bars indicate SE (n = 3). Statistically different (p value < 0.05) is indicated using asterisks.

Furthermore, we detected the plant growth phenotypes under stress conditions. Root growth under salt and mannitol treatment was analyzed, and no significant differences between the mutants and wild type were revealed. However, when *cyp709b3* mutant seedlings were kept on salt plates, more seedlings became bleached and dead. To evaluate the salt tolerance of the *cyp709b3* mutant, the seeds were germinated on MS agar plates for 4 days at which time the seedlings were transferred onto MS agar plates supplemented with 100 mM, 150 mM or 200 mM NaCl. The rate of dead seedlings was scored daily (Figure 4A). When exposed to 150 mM NaCl, more *cyp709b3* seedlings were damaged than wild type (Figure 4B) and other mutants (Figure 4C). From day 5–7, around 70-77% of the *cyp709b3* seedlings were dead under 150 mM NaCl treatment, compared to 26-30% of wild type seedlings (Figure 4A). *cyp709b1* and *cyp709b2* mutants exhibited similar rates of dead seedlings as wild type (Figure 4C). Exposure to 100 mM NaCl caused fewer seedlings to be damaged in both wild type and *cyp709b3* mutants. Under 200 mM NaCl treatment, almost all wild type and *cyp709b3* seedlings were died at day 4. In other words, there was no significant difference between wild type and mutants under mild (100 mM NaCl) or severe salt (200 mM NaCl) treatment.

To further confirm the salt stress phenotype, 12-day-old plants grown in soil were irrigated with 150 mM NaCl. After 2 weeks of treatment, *cyp709b3* mutant plants started showing serious damage compared to wild type and other mutants. As shown in Figure 4D, all plants presented yellowed leaves after 3 weeks of salt treatment; however, *cyp709b3* plants presented more dead plants (fully bleached) under the same growth conditions. There were no differences between any of the genotypes under normal conditions. These results indicate that *cyp709b3* mutant seedlings and plants are more sensitive to moderate salt stress (150 mM NaCl).

### Expression of wild type CYP709B3 gene can rescue the salt sensitive phenotype

**Figure 5:**
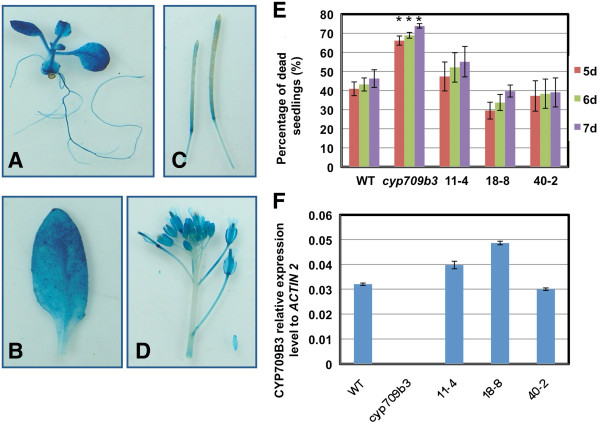
**Expression of wild type CYP709B3 gene in*cyp709b3*mutant can rescue salt intolerance phenotype. A-D.** Expression pattern of *Pro*_*CYP709B3*_*:GUS* in different organs. The results of GUS staining were observed in: seedling **(A)**, leaf **(B)**, silique **(C)** and flower **(D)**. **E.** Expression of a wild type CYP709B3 gene can rescue the salt intolerance phenotype. Wild type and three independent *Pro*_*CYP709B3*_*:CYP709B3* transgenic homozygous lines (11–4, 18–8 and 40–2) in *cyp709b3* mutant background were treated by 150 mM NaCl. The seedlings were counted at the indicated times. **F.** Analysis of the expression level of *CYP709B3* in seedlings from wild type, *cyp709b3* and independent transgenic homozygous lines (11–4, 18–8 and 40–2). *ACTIN2* was used as internal control. Values are the means ± SE of three replicates. Statistically different (p value < 0.05) is indicated using asterisks.

A complementation experiment was performed to further verify the function of CYP709B3. First, the promoter region was obtained by amplifying the 1547-bp region upstream of the ATG start codon. A *Pro*_*CYP709B3*_:*GUS* fusion construct was generated and transformed into wild-type plants. GUS activity was detected in whole seedlings, rosette leaves, siliques and flowers (Figure 5A-D). GUS reporter staining revealed a highly similar expression pattern with this promoter fragment as real-time PCR results did for *CYP709B3* (Figure 2C). Furthermore, a native CYP709B3 promoter construct (*Pro*_*CYP709B3*_*:CYP709B3*) was generated by using the full-length *CYP709B3* genomic DNA that included the same promoter region. When the construct was transformed into the *cyp709b3* T-DNA insertion plants, the wild type gene fully rescued the salt sensitive phenotype displayed in the *cyp709b3* plants (Figure 5E). Three independent homozygous transgenic lines (11–4, 18–8 and 40–2) were selected for detailed analyses. As shown in Figure 5E, these three transgenic lines had similar rates of bleached seedlings as wild type when challenged with150 mM NaCl treatment. The *CYP709B3* gene expression level was similar between the transgenic lines and wild type (Figure 5F). We also found wild type CYP709B3 gene can recue ABA sensitive germination phenotype in transgenic line (Additional file 2). These results strongly support the function of CYP709B3 in salt tolerance.

### Gene expression under salt stress

**Figure 6:**
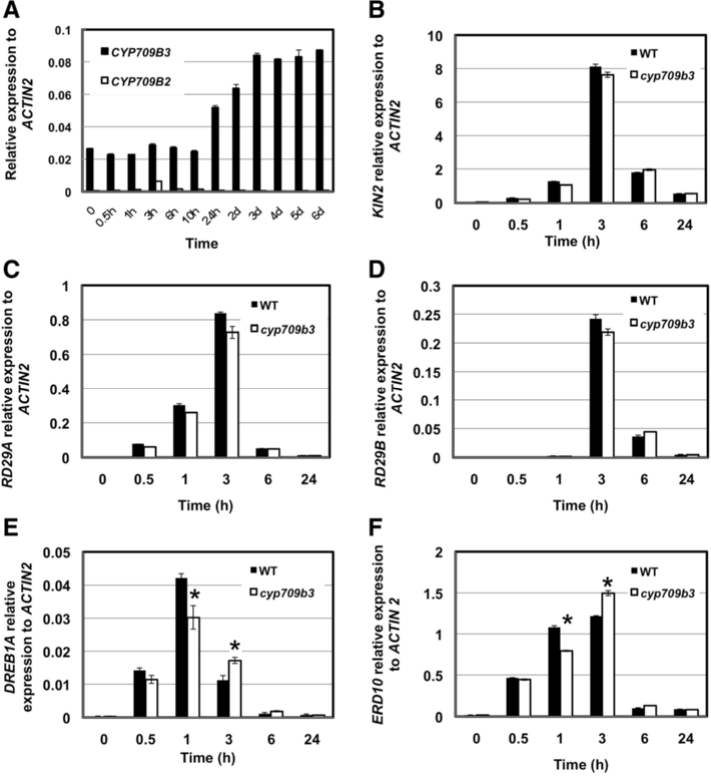
**Gene expression analysis in wild type and mutants under 150 mM NaCl treatment.** Seeds were germinated on MS medium for 4 days and transferred to MS medium supplemented with 150 mM NaCl. Seedlings were harvested at indicated times. Gene expression was detected by quantitative real time PCR. **A.***CYP709B3* and C*YP709B2* gene expression in wild type seedlings under 150 mM NaCl treatment. Expression of stress response genes: *KIN2***(B)**, *RD29A***(C)**, *RD29B***(D)**, *DREB1A***(E)**, *ERD10***(F)** in seedlings from 150 mM NaCl-treated wild type and *cyp709b3* mutant. *ACTIN2* was used as internal control. Error bars indicate SE (n = 3). Statistically different (p value < 0.05) is indicated using asterisks.

The requirement of CYP709B3 in ABA and salt sensitive responses prompted us to investigate whether CYP709B3 was required for stress-regulated gene expression. When *CYP709B3* gene expression under 150 mM NaCl stress was checked, we found that *CYP709B3* gene expression was not induced at the early stage of salt treatment; however, the expression was induced after 24 h and remained high at later time points (Figure 6A). Although CYP709B2 gene expression was dramatically lower than CYP709B3 in seedlings, CYP709B2 gene expression was also induced by salt stress, peaking after 3 hr of 150 mM NaCl treatment and then dropping down to basal level (Figure 6A). CYP709B1 expresses at very low levels in seedlings and was not detected under salt treatment.

Furthermore, we found that stress-regulated genes (*KIN2*, *RD29A* and *RD29B*) in the *cyp709b3* mutant were not significantly altered compared to wild type. *DREB1A* and *ERD10* expression was slightly decreased at 1 hour after 150 mM NaCl treatment (Figure 6B-F), indicating that the *cyp709b3* mutant did not substantially impair the up-regulation of stress-regulated genes.

### ABA content is not affected in the *cyp709b3* mutant

**Figure 7:**
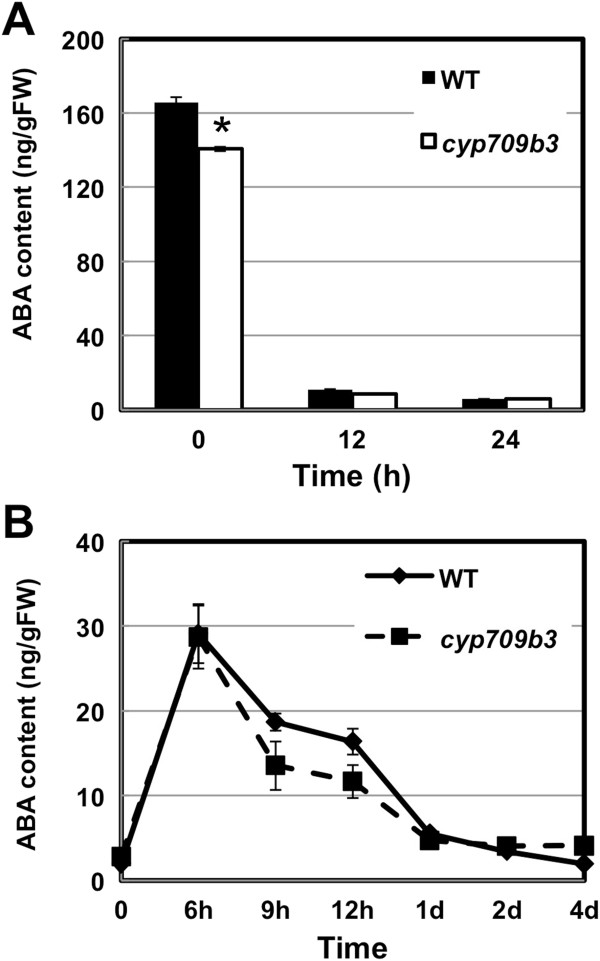
**The endogenous level of ABA in wild type and mutants. A.** The change of endogenous ABA levels in wild type and *cyp709b3* mutant during seed imbibition. The seeds were collected at 0, 12 and 24 h after imbibition. **B.** Endogenous ABA level in seedlings from 150 mM NaCl-treated seedlings from wild type and *cyp709b3* at indicated times. Values are the means ± SE of three replicates. Statistically different (p value < 0.05) is indicated using asterisks.

The phytohormone ABA is key in regulating plant stress responses and plays important roles in seed germination [24]. Since the *cyp709b3* mutant seed is sensitive to both ABA and salt in germination, we analyzed the endogenous ABA content during seed imbibition and salt stress. During seed imbibition, the change in the endogenous ABA level in the *cyp709b3* mutant is the same as in wild type seeds (Figure 7A). Endogenous ABA content decreased to the same levels 12 and 24 hours after imbibition in both *cyp709b3* mutant and wild type. After 150 mM NaCl treatment, the ABA content reached a similar maximal level in both wild type and *cyp709b3* seedlings at 6 hours*,* then dropped down to the basal level 2 day after treatment (Figure 7B). These data clearly demonstrate that CYP709B subfamily genes are not involved in ABA metabolism.

### Metabolite profiling under salt stress

Metabolite profiles of wild type and the *cyp709b3* mutant under both normal and salt stress growth condition were determined using global metabolomic analysis (METABOLON Inc.).

All wild type and *cyp709b3* seedlings were green for the first 3 days after transferring onto 150 mM NaCl plates. From day 4 onwards, the *cyp709b3* mutant developed more bleached and dying seedlings than wild type (Figure 4). Both wild type and *cyp709b3* seedlings grew normally on MS plates without salt. Therefore, we collected seedling samples at day 2 (2D) and day 4 (4D) from non-salt treated (N) and salt treated (S) plates. Some seedlings were already dead at day 4; we did not pick up these dead seedlings for metabolite analysis. All extracted samples were analyzed by LC/MS and GC/MS. The identified 163 metabolites contain amino acids, carbohydrates, lipids, cofactors, prosthetic groups, electron carriers, nucleotides, peptides, hormones and secondary metabolites.

The metabolomes of both wild type and *cyp709b3* were strongly affected by the salt treatment; however, the differences between the lines at similar treatments were relatively subtle. Of the 163 compounds tested, there were no obvious indications of a substrate or product whose presence was associated absolutely with the presence of the mutation (Additional file 3). Nonetheless, some trends did suggest biologically relevant genotype-related differences, both in the absence and presence of salt stress. Of the 163 compounds tested, 61-75% differed significantly (*p* < 0.05) in the non-salt (N) *vs.* salt (S) tests. This is in sharp contrast to the time-related differences within either line (2D *vs*. 4D), in which 23-33% of the compounds were different and especially to the genotype-related differences from 6-25% within treatments (MUT *vs*. WT) (Additional file 4).

The very large number of compounds altered by salt stress illustrates the profound effect of salt stress on *Arabidopsis* seedlings. Many of these changes were greater than 5-fold in magnitude, and they included compounds expected to be induced during salt stress (proline and histidine, among many amino acids, glutathione, and GABA).

More general metabolomic perturbations were observed in the non-stressed mutant plants. These include several indications of oxidative and ammonia stress. For example, NAD^+^ and dehydroascorbate, two compounds in pathways which supply intermediates for the remediation of oxidative stress, were lower in non-stressed mutant plants, while ophthalmate, *gamma*-glutamylglutamate, and beta-alanine were higher.

The large number of strong changes of metabolites tends to overpower any mutant effects. Almost all affected compounds behaved in a similar manner between WT and mutant, in both direction and magnitude of change. However, a few exceptions can be noted. Mutant plants at Day 4 were not able to maintain the induction of spermidine and pantothenate, which could lead to a shortage of CoA, an important cofactor in a wide range of reactions.

**Figure 8:**
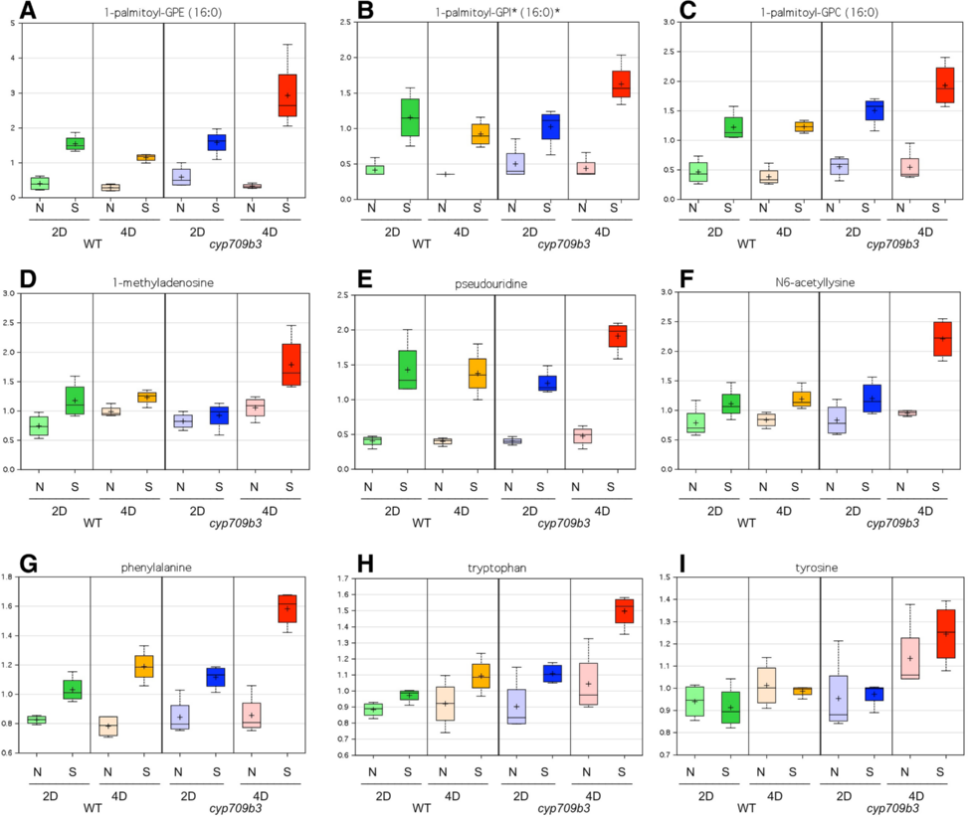
**Comparison of metabolite profiling between wild type and*cyp709b3*.** Compounds related to membrane degradation **(A-C)**, nucleic acid **(D-F)** and protein degradation **(G-I)** was increased in *cyp709b3* mutants under salt stress compared to wild type (WT). Four-day-old seedlings were transferred onto 150 mM NaCl plates. Untreated and treated seedlings were collected at 2 days (2D) and 4 days (4D) after treatment. 100 mg of tissue was extracted and analyzed by LC/MS and GC/MS by Metabolon, Inc. Values are the means ± SD of four replicates. N: non-salt treatment; S: salt treatment. Y-axis: peak intensity.

Significant changes in several compounds indicate increased cellular damage in the *cyp709b3* plants. While these compounds respond to salt stress in a similar direction in both WT and mutant, the magnitude of the response is significantly stronger in the mutant. For example, the lysolipids are markers of lipolysis in membranes and of membrane damage. All three 1-palmitoyl-lysolipids showed a similar pattern of stronger induction in *cyp709b3* 4D salt-treated plants (Figure 8A, B and C). Likewise, 1-methyladenosine and pseudouridine are markers of nucleic acid turnover; both these compounds are modified post-transcriptionally, and thus represent macromolecular degradation. As shown in Figure 8D and E, both accumulated in the *cyp709b3* mutant at 4D after salt treatment. N6-acetyllysine, a product of protein breakdown, also increased in treated *cyp709b3* 4D samples (Figure 8F). Additionally, all the aromatic amino acids exhibited similar patterns of increased salt-response, which may reflect protein turnover (Figure 8G, H and I). These results indicate that some changes of metabolism under salt stress are related to the *cyp709b3* salt intolerance phenotype.

## Discussion

In *Arabidopsis*, a minority of the P450s have been characterized in detail, with the biochemical function of only a few being fully elucidated [7,10]. More than 200 genes remain uncharacterized. Several approaches are being used to study these unknown genes, such as expression and co-expression analysis, genetic screening, and heterologous expression assays.

### *CYP709B3* plays roles in salt tolerance

Gene expression patterns identify genes with correlated functions during plant development, or in response to various stimuli. Recently, gene expression profiling and co-expression analysis of the cytochrome P450 superfamily in *Arabidopsis* was analyzed using cDNA microarrays [9,11]. The expression profiling of the CYP709B subfamily was reported in several publications. For example, Duan et al. compared the gene expression profiles of flower and leaf tissues of both Col and L*er Arabidopsis* ecotypes using P450 microarrays [25]. They found that *CYP709B2* gene expression in flowers is higher in the L*er* ecotype than in the Col ecotype. Furthermore, some of the gene members in the CYP709B subfamily can be regulated by phytohormones: Auxin up-regulates *CYP709B2* expression and brassinosteriod down-regulates *CYP709B3* expression at later times after treatment [19]. In another study, expression of the *CYP709B3* gene showed circadian regulation [20].

In this report, we detected the expression patterns of the three members of the CYP709B subfamily. The results revealed different expression patterns of the genes in the various organs examined. *CYP709B3* was expressed universally, but was expressed at the highest levels in leaves and siliques. *CYP709B1* and *CYP709B2* were highly expressed in siliques but weakly expressed in other examined organs (Figure 2). Furthermore, we found that *CYP709B3* expression can be induced by salt stress and continually induced after 24 h of treatment. While expression of *CYP709B2* in seedlings is very low, it was also induced by salt stress. In these experiments, *CYP709B1* expression was not detected in either the salt-treated or untreated tissues. These expression profiles indicate that the three *CYP709B* genes may have divergent functions in plant development or stress response.

Several research groups have tried to identify the enzymatic functions of CYP709B subfamily members using heterologous expression systems. Using an *adenosine phosphate-isopentenyltransferase* (*AtIPT4*)*/P450* co-expression system in yeast, the CYP735A (formerly named CYP709A) subfamily was identified as a cytokinin hydroxylase that catalyzes the biosynthesis of *trans*-Zeatin. Phylogenetic analysis of *Arabidopsis* P450 genes shows that the CYP709B subfamily is a sister group to the CYP735A subfamily. However, no hydroxylase activity was detected for the CYP709Bs in the yeast system [14]. Kandel et al*.* characterized CYP709C1 from wheat (homolog of the CYP709Bs) as an in-chain hydroxylase [21,22]. Although they tried to detect the same enzymatic activity in the CYP709B subfamily, expression of CYP709B1, CYP709B2, or CYP709B3 in yeast failed to demonstrate any in-chain hydroxylase activity.

To further investigate the functions of the CYP709B subfamily members, we identified T-DNA insertional null mutants. None of the null mutants had a visible morphological alteration, indicating that the CYP709B genes were not essential to plant development. Only the *cyp709b3* mutant showed ABA and salt sensitivity in germination (Figure 3) and deficiency in salt tolerance (Figure 4), indicating that CYP709B3 has a unique function in ABA and salt stress responses that is not shared by CYP709B1 and CYP709B2.

### CYP709B3 is not directly involved in ABA metabolism or up-regulation of stress-regulated genes in plants

Abscisic acid (ABA) plays pivotal roles in many cellular processes, including seed development, dormancy, germination, vegetative growth and environmental stress response. Environmental stresses can dramatically increase ABA levels. The increased ABA levels under stress are due to both active ABA biosynthesis and suppressed ABA degradation. Recently, several published papers have shown that the CYP707A subfamily is involved in ABA metabolism [15]. In the *Arabidopsis* genome, there are four CYP707A genes; *CYP707A1-4*. Expression of *CYP707A2* is specifically up-regulated when *Arabidopsis* seeds are imbibed, and thus rapidly depletes the ABA pool and releases the seeds from dormancy. Seeds of the mutant *cyp707a2* exhibited hyperdormancy and accumulated six-fold greater ABA content than wild type. Expression of all four *CYP707A* genes was increased when stressed leaves were rehydrated. Thus, it is clear that expression of the *CYP707A* genes play a key role in regulating ABA levels. Furthermore, recombinant CYP707A2 protein exclusively oxidized ABA to 8′-hydroxy-ABA and PA. More data shows that other CYP707A genes also have 8′-hydroxylase activity [16-18]. Since both of CYP709B and CYP707A subfamilies are non-A-type cytochrome P450s, and *cyp709b3* mutant shows ABA and salt sensitive phenotype, we speculated that the CYP709B subfamily may be involved in ABA catabolism. To address this question, we measured ABA content in seeds and seedlings of wild type and *cyp709b3* mutant. As shown in Figure 7, the ABA content was similar between wild type and *cyp709b3* mutant during seed imbibition and salt treatment. These results rule out a role for the CYP709B3 protein in ABA catabolism during seed germination or salt stress responses.

In order to elucidate the molecular mechanism governing the salt intolerance phenotype in *cyp709b3*, we analyzed the expression of ABA and stress-induced marker genes under 150 mM NaCl treatment. The transcript levels of representative stress-regulated genes (*KIN2, RD29A*, *RD29B, DREB1A* and *ERD10*) in the *cyp709b3* mutant were not significantly altered relative to the wild type under salt stress (Figure 6B, C, D, E and F). These data suggest that the *cyp709b3* mutant did not impair the up-regulation of these genes by salt stress. Since ABA content and the expression of stress up-regulated marker genes was not affected in the *cyp709b3* mutant compared to WT under salt treatment, we therefore conclude that CYP709B3 is not the primary gene involved in ABA signaling or biosynthesis under salt stress. ABA content and the expression of stress up-regulated marker genes reached their highest level at an early time point (before 6 hours); however, the CYP709B3 gene did not increase expression until 24 hours. We therefore suggest that CYP709B3 plays a role in the later stage of salt tolerance. We also searched the expression pattern of CYP709B3 in ABA signaling (*abi4-102* and *abi1-1*) and synthesis deficient mutants (*aba1-1*) from published microarray data (http://www.genevestigator.com). CYP709B3 expression is not significantly different between ABA mutants and wild type (Additional file 5). According to these results, we conclude that there is not a close relationship between CYP709B3 and ABA signaling or synthesis. Therefore, CYP709B3 may regulate the salt stress response through a novel pathway independent of the well-characterized regulators.

### Is CYP709B3 involved in salt stress response through alteration of metabolic process?

When plants are challenged with hyperosmolarity, the osmotic potential in the cell will be increased. To solve this problem, plant cells will accumulate compatible osmolytes to decrease the osmotic potential of the cell cytosol and preserve the activity of enzymes in saline solutions [26]. Various compatible osmolytes, such as proline, polyamines, organic acids and glycine betaine, can greatly reduce stress damage to plant cells [27-32]. The synthesis of compatible osmolytes is often achieved by diversion of basic intermediary metabolites into these unique biochemical reactions, a diversion that is often triggered by stress. In general, cytochrome P450s are involved in primary and secondary metabolism and may be involved in the biosynthesis of some osmolytes. Recently, metabolite profiling analysis was preformed to study metabolic responses to stress in plants [33,34]. Other potential biochemical compounds involved in salt tolerance can be identified by metabolic analysis [35-37]. In this report, metabolite profiling analysis was performed to compare the differences in metabolism between wild type and *cyp709b3* under normal and salt stress conditions, and to further identify the substrates of CYP709B3. The initial focus was on compounds whose synthesis or degradation might involve P450 enzymes, which are typically involved in hydroxylation and other oxidative reactions. The metabolomes of both wild type and *cyp709b3* were strongly affected by the salt treatment (Additional files 3 and 4); however, the differences between the lines subjected to similar treatments were relatively subtle. For example, the osmolyte proline had a strong salt-stress response that was similar in direction and magnitude between wild type and *cyp709b3* (Additional file 6). Some compounds related to membrane degradation and nucleic acid and protein degradation were increased in *cyp709b3* under salt stress compared to wild type (Figure 8), further confirming the salt intolerance response of the *cyp709b3* mutant. So far, we did not find changes in compounds that would be expected to be synthesized through P450 enzyme activity (hydroxylation and oxidation). We speculate that CYP709B3 possesses a specific, unidentified enzyme activity that produces a biochemical compound that regulates salt stress response. Due to the limited number of compounds tested in this report (163 known compounds), further analysis of unknown compounds will provide the information needed to identify the substrate (or substrates) of CYP709B3 and its function in salt tolerance.

## Conclusions

The *cyp709b3* null mutant shows an ABA sensitive and salt intolerance phenotype. Expression of the wild type CYP709B3 gene in the *cyp709b3* mutant fully complemented the salt intolerance phenotype. The expression of CYP709B3 gene is induced by salt stress. These data demonstrate that the CYP709B3 gene plays a role in the regulation of salt tolerance in *Arabidopsis*. Further analysis indicates that CYP709B3 may regulate the salt stress response through an unknown pathway independent of the well-characterized regulator.

## Methods

### Plant materials, mutants screening and statistical analysis

Mutant and wild-type plants in *Arabidopsis thaliana* ecotype Columbia (Col-0) were used in all experiments. Plants were grown under long day conditions (16 h light/8 h dark) with about 125 μE m^-2^ s^-1^ light at 22°C. T-DNA insertion mutants of *CYP709B1* (At2g46960), *CYP709B2* (At2g46950) and *CYP709B3* (At4g27710) were obtained from the Arabidopsis Biological Resource Center [38,39]. Homozygous null mutants were screened by genomic PCR using gene-specific primers. The primer pairs used for identification of mutants are 5′- gtcaggtgcgttgaaaacttg-3′ and 5′- tgagatgcatatccttggctc-3′ for *cyp709b1*, 5′- actcgttagagcttgcagctg-3′ and 5′- ctcctgagcacgatcaatctc-3′ for *cyp709b2-1,* 5′- ttgtgagacgatcacgtgaac-3′ and 5′- gtcgctatgatatcagcggtc-3′ for *cyp709b2-2,* and 5′- catgagctagcgaaacaggtc-3′ and 5′- tttaatcacgggtccgtacag-3′ for *cyp709b3.*

For observation of seedling phenotypes, sterilized seeds were plated on a half-strength Murashige and Skoog (MS) medium with 1% sucrose and 0.6% agar. Plates were incubated in a growth chamber at 22°C under continuous light (100 μE m^-2^ sec^-1^). Seedlings were grown in different treatment conditions for phenotypic investigation.

At least two biological repetitions were performed for all experiments. For single experiments, at least three independent repetitions were done. Results from one of the biological repeats that gave comparable results were shown. Student’s t test was used to determine whether the difference between two groups of data at a specific time point is statistically significant (P < 0.05). Statistically different data groups are indicated in the relevant figures.

### Germination and stress tolerance assays

For germination assays, around 100 seeds each from wild type and mutants were sown in triplicate on filter paper soaked with distilled water, or with different concentrations of ABA, or on MS plates with the addition of 0, 100, 150 or 200 mM NaCl. Seed germination (emergence of radicals) was scored daily. For the salt tolerance assay, around 80 wild type and mutant seeds were germinated on MS agar plates. After 4-days growth, seedlings were transferred onto MS agar plates with addition of different concentrations of NaCl. The seedlings with fully yellow and bleached cotyledon were scored as dead seedlings. Only seedlings that developed true leaves and remained green were scored as survival seedlings. Seedlings were collected at indicated times for real time PCR and phytohormone analysis. Each treatment was performed in triplicate [40].

### RNA extraction and real time PCR

Total RNA was extracted using TRIzol reagent (Invitrogen). After DNase treatment (Turbo DNA-free, Ambion), 1 μg of total RNA was used for reverse transcription. Quantitative real time PCR assays were performed using the generated cDNA as template and gene-specific primers. Semi-quantitative RT-PCR was performed to detect transcript levels in wild type and mutants. The primer pairs were 5′- atgttgtcgaacaagttaggtttc -3′ and 5′- gttatccacaggggtgtgct -3′ for *CYP709B1*, 5′- cgaccctcacactacacacg-3′ and 5′- cggaaccgttgaagaatcat-3′ for *CYP709B2,* 5′- atggaacttataagcacaatcaatctc-3′ and 5′- atactcgggggagaggctaa-3′ for *CYP709B3,* and 5′- cactgtgccaatctacgagggt -3′ and 5′- cacaaacgagggctggaacaag -3′ for *ACTIN2.*

Real time PCR was conducted using SYBR Green Fast Mix Rox (Quanta) on a StepOnePlus Real-Time PCR System (Applied Biosystems). Estimates of transcript amount were performed using the comparative threshold cycle method. Relative expression levels were normalized using *ACTIN2* as an internal control. The primer pairs (forward and reverse) used for real time PCR were *ACTIN2* (At3g18780, 5′- ggtaacattgtgctcagtggtgg-3′ and 5′- aacgaccttaatcttcatgctgc-3′), *CYP709B1* (At2g46960, 5′- ggcatccatttgtgttgaccagaag-3′ and 5′- cttctttatctcgctgaggtttccg-3′), *CYP709B2* (At2g46950, 5′- atgcacagagacaaagccgtttgg-3′ and 5′- ccaatgcaagctcttggtcccatt-3′), *CYP709B3* (At4g27710, 5′- tgttgatggagtcgcttcgtctgt-3′ and 5′- tggccttgtctctgtgcatcttca-3′), *RD29A* (At5g52310, 5′- gttactgatcccaccaaagaaga-3′ and 5′- ggagactcatcagtcacttcca-3′), *RD29B* (At5g52300, 5′-acaatcacttggcaccaccgtt-3′ and 5′- aactacttccaccggaatccgaa-3′) , *KIN2* (At5g15970, 5′- gcaacaggcgggaaagagtat-3′ and 5′- ccggtcttgtccttcacgaa-3′), *DREB1A* (At4g25480, 5′-gatcagcctgtctcaatttc-3′ and 5′-cttctgccatattagccaac-3′)and *ERD10* (At1g20450, 5′-tctctgaaccagagtcgttt-3′ and 5′-cttcttctcaccgtcttcac-3′)

### Quantitative measurement of ABA

A method used for detection and quantification of acidic plant hormones was developed and performed by the Proteomics & Mass Spectrometry Facility at the Donald Danforth Plant Science Center. The method was modified according to published reference [41].

### Plasmid constructs and plant transformation

To make the *CYP709B3* complementation construct, the full-length *CYP709B3* genomic DNA, including the 1547-bp region upstream of the ATG start codon, was PCR-amplified using CYP709B3-Pro-F (5′-ccaaagaaagcaaagccaag-3′) and CYP709B3-R (5′-tccgagagggtgaagcattacg-3′) primers. The fragment was cloned into the pCR8/GW/TOPO (Invitrogen) vector. The LR recombination reaction was performed to transfer the fragment to the plant expression vector pMDC99 [42] in order to generate the final construct *Pro*_*CYP709B3*_*:CYP709B3*. For the GUS fusion construct, the 1547-bp region upstream of the ATG start codon was amplified by PCR using CYP709B3-Pro-F and CYP709B3-Pro-R (5′- taaaagaaggaacacaagtagctc-3′) primers and introduced into pCR8/GW/TOPO. Finally, a LR recombination reaction was performed to transfer the fragment to the plant expression vector pMDC162 in order to generate the final construct of *Pro*_*CYP709B3*_*:GUS.* All constructs were sequence confirmed. All constructs were introduced into *Agrobacterium tumefaciens* strain GV3101 and then transformed into wild-type or *cyp709b3* mutant plants by the floral dipping method [43].

### GUS staining assay

GUS staining was performed according to a published method [44].

### Metabolite profiling analysis

Wild type (W) and *cyp709b3* (M) seedlings were collected after 2 days (2D) and 4 days (4D) of growth on normal or 150 mM NaCl MS plates. Sample extraction, LC/MS and GC/MS analysis were performed as described [34] by METABOLON, Inc. In brief, 100 mg of each sample were extracted using the automated Microlab STAR system. Recovery standards were added prior to the first step in the extraction process. Sample preparation was conducted using a proprietary series of organic and aqueous extractions to remove the protein fraction while allowing maximum recovery of small molecules. The resulting extract was divided into two fractions; one for analysis by LC/MS and one for analysis by GC/MS. Samples were placed briefly on a TurboVap® (Zymark) to remove the organic solvent. Each sample was then frozen and dried under vacuum. Samples were then prepared for the appropriate their instrument, either LC/MS or GC/MS.

## Abbreviations

PCR: Polymerase chain reaction; LC/MS: Liquid chromatography-mass spectrometry; GC/MS: Gas chromatography–mass spectrometry.

## Competing interests

The authors have declared no conflict of interests.

## Authors’ contributions

GM and OY conceived the study, designed the experiments and drafted the manuscript. GM, TS and DS performed the experiments. All authors read and approved the final manuscript.

## Supplementary Material

Additional file 1 **Amino acid alignment of CYP709B1, CYP709B2 and CYP709B3.** The analysis was performed using ClustalW2. Click here for file

Additional file 2 **CYP709B3 gene can rescue ABA sensitive phenotype in seed germination.** Seeds were sown on wetted filter paper containing 0 μM ABA (A) and 1.5 μM ABA (B). After 2 days at 4°C, the plates were placed under continuous light. Germination (emergence of radicals) was scored at indicated times. Error bars indicate SE (n = 3). Statistically different to wild type (p value < 0.05) is indicated using asterisks. Click here for file

Additional file 3 **Metabolomic difference between wild type and *cyp709b3, *with and without salt treatment. Four-day-old seedlings were transferred onto 150 mM NaCl plates.** Untreated and treated seedlings were collected at 2 days (2D) and 4 days (4D) after treatment. 100 mg of tissue was extracted and analyzed by LC/MS and GC/MS by Metabolon, Inc. Values are the means ± SD of four replicates and presented as ratios. NS: non-salt treatment; SALT: salt treatment. Heat map of statistically significant biochemicals profiled in this study. Shaded cells indicate p ≤ 0.05 (red indicates that the mean values are significantly higher for that comparison; green values significantly lower). Click here for file

Additional file 4 **Comparison of metabolites between wild type (WT) and *cyp709b3 *** **(MUT) under non-salt (N) and salt (S) conditions at day 2 (2D) and day 4 (4D).** Click here for file

Additional file 5 **CYP709B3 gene expression in ABA signaling and ABA biosynthesis deficient mutants.** From http://www.genevestigator.com. Click here for file

Additional file 6 **Proline analysis in seedling samples.** Four-day-old seedlings were transferred onto 150 mM NaCl plates. Untreated and treated seedlings were collected at 2 days (2D) and 4 days (4D) after treatment. 100 mg of tissue was extracted and analyzed by LC/MS and GC/MS by Metabolon, Inc. Values are the means ± SD of four replicates. N: non-salt treatment; S: salt treatment. Y-axis: peak intensity. Click here for file
