# Supplementary figures and images for: CYP709B3, a cytochrome P450 monooxygenase gene involved in salt tolerance in Arabidopsis thaliana
Source: BMC Plant Biol. 2013 Oct 28;13:169. doi: 10.1186/1471-2229-13-169 (PMC3819737; doi:10.1186/1471-2229-13-169)

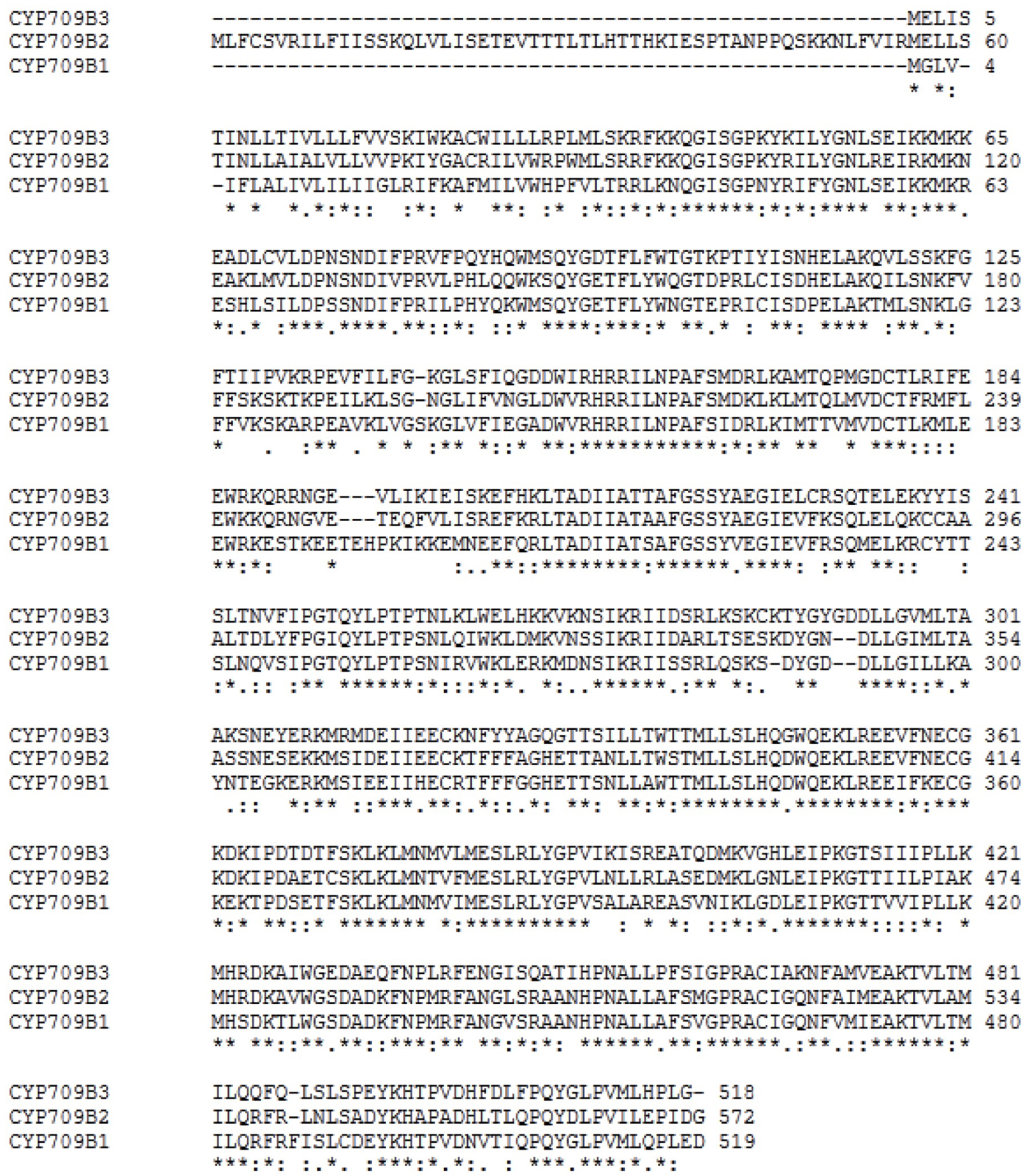

Supplement: Additional file 1 — Amino acid alignment of CYP709B1, CYP709B2 and CYP709B3. The analysis was performed using ClustalW2. [file 1471-2229-13-169-S1.jpeg]

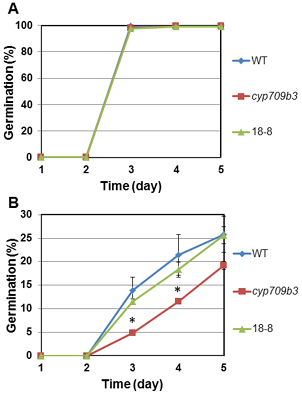

Supplement: Additional file 2 — CYP709B3 gene can rescue ABA sensitive phenotype in seed germination. Seeds were sown on wetted filter paper containing 0 μM ABA (A) and 1.5 μM ABA (B). After 2 days at 4°C, the plates were placed under continuous light. Germination (emergence of radicals) was scored at indicated times. Error bars indicate SE (n = 3). Statistically different to wild type (p value < 0.05) is indicated using asterisks. [file 1471-2229-13-169-S2.jpeg]

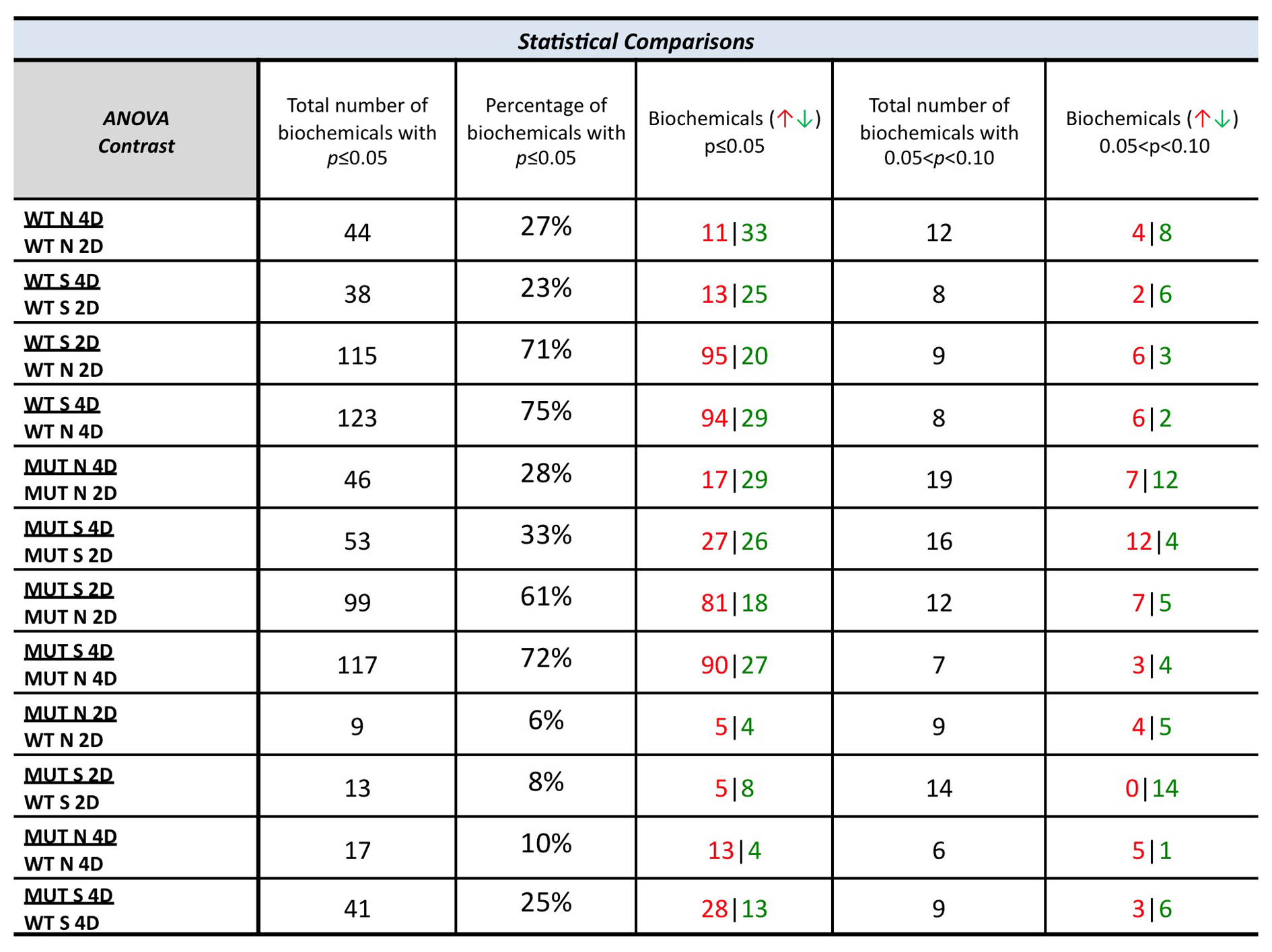

Supplement: Additional file 4 — Comparison of metabolites between wild type (WT) and cyp709b3 (MUT) under non-salt (N) and salt (S) conditions at day 2 (2D) and day 4 (4D). [file 1471-2229-13-169-S4.jpeg]

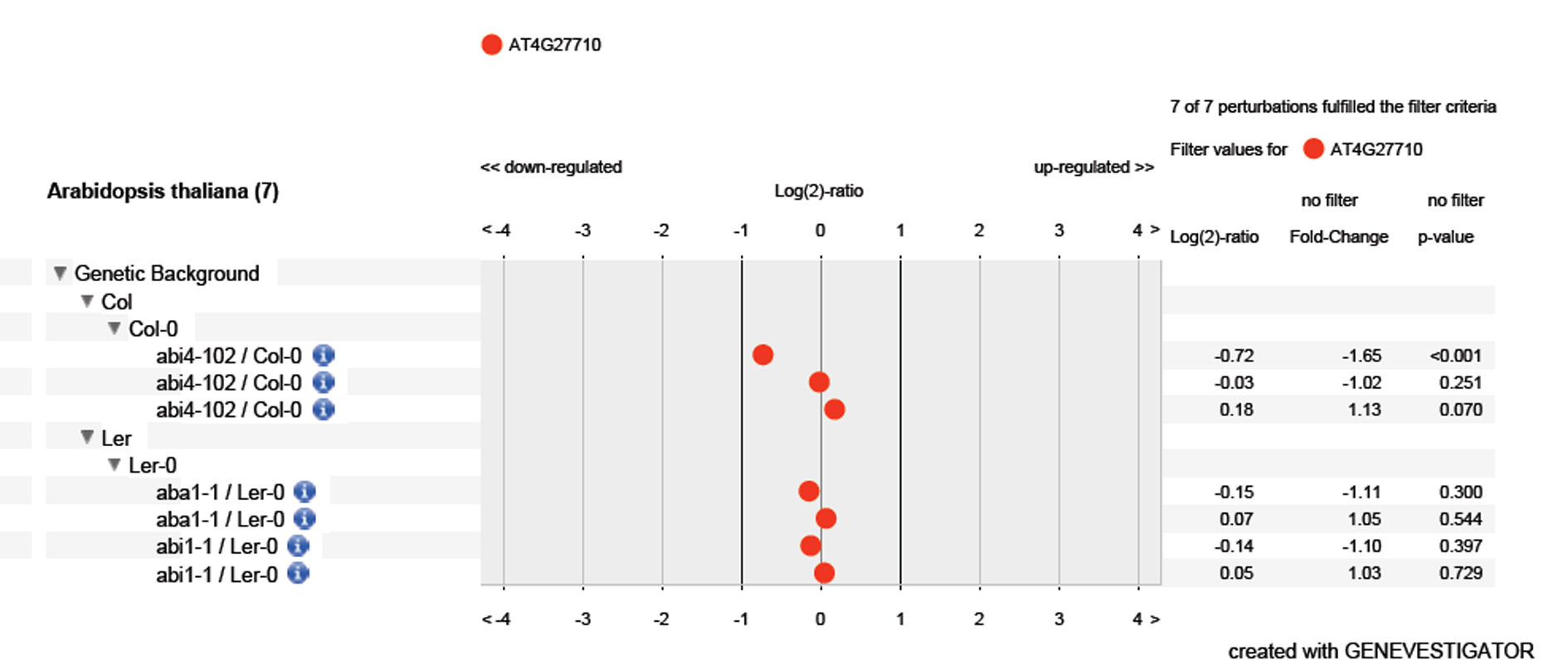

Supplement: Additional file 5 — CYP709B3 gene expression in ABA signaling and ABA biosynthesis deficient mutants. From http://www.genevestigator.com. [file 1471-2229-13-169-S5.jpeg]

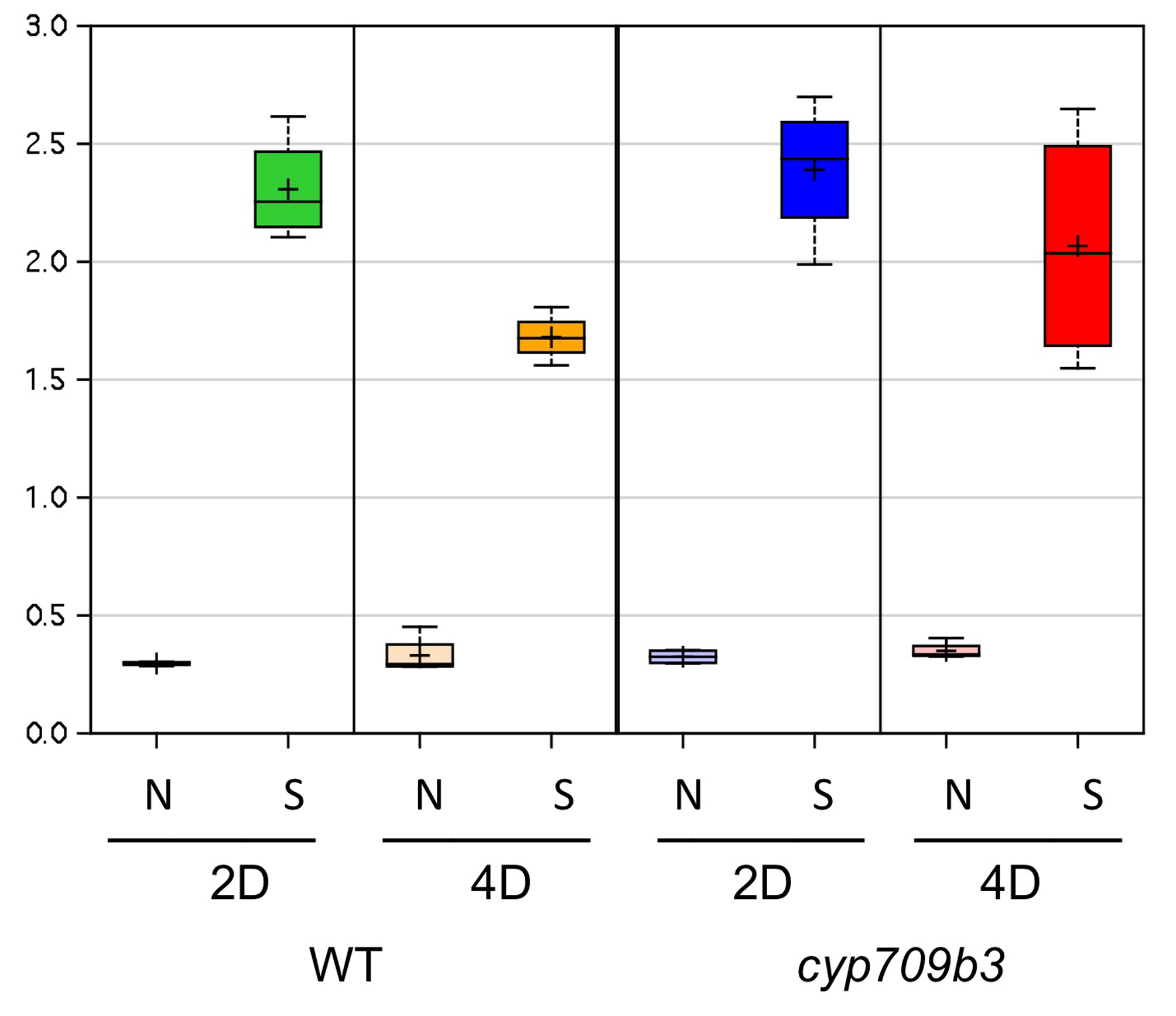

Supplement: Additional file 6 — Proline analysis in seedling samples. Four-day-old seedlings were transferred onto 150 mM NaCl plates. Untreated and treated seedlings were collected at 2 days (2D) and 4 days (4D) after treatment. 100 mg of tissue was extracted and analyzed by LC/MS and GC/MS by Metabolon, Inc. Values are the means ± SD of four replicates. N: non-salt treatment; S: salt treatment. Y-axis: peak intensity. [file 1471-2229-13-169-S6.jpeg]
